# Supplementary material for: Genome-wide characterization and expression analysis of PP2CA family members in response to ABA and osmotic stress in Gossypium
Source: PeerJ. 2019 Jun 14;7:e7105. doi: 10.7717/peerj.7105 (PMC6573834; doi:10.7717/peerj.7105)
Supplement: Supplemental Information 1 — Primer Premier 5 software was used to design the primers depending on the CDS of the GhPP2CA genes. The lengths of amplified fragment ranged from 80 bp to 300 bp. [file peerj-07-7105-s001.docx]

Table S1 Gene primers used for quantitative real-time RT-PCR experiments

| Genes | Gene number | Forward primers (5ʹ-3ʹ) | Reverse primers (5ʹ-3ʹ) |
| --- | --- | --- | --- |
| *GhUBQ7* | Gh_A11G0969 | GAAGGCATTCCACCTGACCAAC | CTTGACCTTCTTCTTCTTGTGCTTG |
| *GhPP2CA1* | Gh_A03G0373 | TTCCGTTATCGTCTGACCACA | GACATTGCTAGGACTCCGAGG |
| *GhPP2CA2* | Gh_A05G0308 | ATATCTGACAGTAAAGATGTTTGG | AAAAAATCCTCTCCGCAAA |
| *GhPP2CA3* | Gh_A05G0782 | AAGCTGAGAATGGCCGG | ACAGCGTTTTCACAGTCCAG |
| *GhPP2CA4* | Gh_A05G1136 | TGGAGGAAAAGTTAGCCAAG | ACCACAGTTAGCAACAACGATA |
| *GhPP2CA5* | Gh_A05G3030 | GTGGAGTGGAAGCGGACG | CGGCAACTTGAACGCTCC |
| *GhPP2CA6* | Gh_A06G0579 | GCGTACTAAGACGGCGGC | CCTTCTTCAATCTCCACTGCC |
| *GhPP2CA7* | Gh_A07G0123 | TTCCCAATCCAGAAGTAATGTT | GGTCAATTCCTTCACCTCTTTC |
| *GhPP2CA8* | Gh_A08G2192 | ATGCCGAGAGCATATCGA | GCCAGTCGTATCTGATGTTTC |
| *GhPP2CA9* | Gh_A10G0578 | AGGAGCTTTGCGGAATGGT | ATCTGGCAGGGTGAGACAATAA |
| *GhPP2CA10* | Gh_A10G1998 | GAAGCTGGTGGCCGAGTAAT | CATAGGTGGTGATGCTGGCT |
| *GhPP2CA11* | Gh_A12G2380 | GCTCACAAGAGGCTGCG | CATCCACAATGGTGTCCCAC |
| *GhPP2CA12* | Gh_A13G0184 | TCACTTTTTCGGTGTCTTCG | CTCTAACTTCTTCATCCATCCTCT |
| *GhPP2CA13* | Gh_A13G1741 | CATTGACTGTAGATCAGAAGCC | TCGCTGGCTATTACTAAACACT |
| *GhPP2CA14* | Gh_D03G1169 | CGTTGGCTTCCACTATTTTG | ATCCCATAAACCGTCACTCG |
| *GhPP2CA15* | Gh_D04G0612 | GTGGACTGGAAGGGGACGA | CTCATCAGGCGTAACGACAGC |
| *GhPP2CA16* | Gh_D05G0410 | TTGCCGTGCCAGTAAACCT | TCGAGAGCCAATTGAAGAACC |
| *GhPP2CA17* | Gh_D05G1309 | TGATGAGATCGGAGGAAAAAT | ACAACGATATGCGATGAACA |
| *GhPP2CA18* | Gh_D05G3907 | GGGAGGTCGGGTCATTTTC | AACCGTCACTCGCCAGAATC |
| *GhPP2CA19* | Gh_D06G0657 | CAGAGGAATGGGGGCGT | CTACAGCAGCGGTGTGGAC |
| *GhPP2CA20* | Gh_D07G2383 | CTCGATTTTTGCAAGTCCCTA | CCTTTCTATCTCCTCGGCTAA |
| *GhPP2CA21* | Gh_D08G2557 | CTGAATGTCGAGAGCGTATCG | CCCGCCAGTCGTATCCG |
| *GhPP2CA22* | Gh_D10G0622 | AACCATTTGATGTAAGAAGGATTT | CATTGTCAGCCCTCCCATA |
| *GhPP2CA23* | Gh_D10G2305 | GAAGCGATGGACTATGGGA | GACATAGGTGGTGATGCTGG |
| *GhPP2CA24* | Gh_D12G2508 | CCGATTGTATCATCAGAACCC | GAACATCCCATAAACCATCACTT |
| *GhPP2CA25* | Gh_D13G0199 | TCTCGGGATTGTGATAACGC | CGATCTCTACACTTCATCGCAAC |
| *GhPP2CA26* | Gh_D13G2089 | GCATTAACTGTTGATCAGAAGCC | ACATTACATCCCATAACCCGTC |
| *GhPP2CA27* | Sca051315G01 | CATCAAGTGGAACGAGAGCG | CATTGCGAGGACACCGAGA |
