# Supplementary material for: Genome-wide characterization and expression analysis of PP2CA family members in response to ABA and osmotic stress in Gossypium
Source: PeerJ. 2019 Jun 14;7:e7105. doi: 10.7717/peerj.7105 (PMC6573834; doi:10.7717/peerj.7105)
Supplement: Supplemental Information 2 — Primer Premier 5 software was used to design the primers to amplify the CDS fragments of GhPP2CA genes.. [file peerj-07-7105-s002.docx]

Table S2 Gene primers applied in yeast two-hybrid experiments

| Genes | Gene number | Forward primers (5ʹ-3ʹ) | Reverse primers (5ʹ-3ʹ) |
| --- | --- | --- | --- |
| *GhPP2CA2* | Gh_A05G0308 | CCGGAATTCATGGAGGAGATGTCTTTGAC | GACGTCGACGGTCTTACTCTTGAACTTCC |
| *GhPP2CA3* | Gh_A05G0782 | CCGGAATTCATGGCGGAGATCTATCGCGG | GCACTGCAGCGTGGCTCTCTTTAGATCCACCAC |
| *GhPP2CA6* | Gh_A06G0579 | CCGGAATTCATGGAACCTCCACGGGAAGA | GCACTGCAGGTTGAAGAGAAGGGATTGGATTAGGT |
| *GhPP2CA10* | Gh_A10G1998 | CCGGAATTCATGGCTGGAGTTTGCTGTGGA | GCACTGCAGTTGATTTTCCTTCAAATCAACAACGACAA |
| *GhPP2CA13* | Gh_A13G1741 | CCGGAATTCATGGAACCCCTACTTGAAGAAG | GACGTCGACTCTTTTTTTCACCGCATTGGAT |
| *GhPP2CA15* | Gh_D04G0612 | CCGGAATTCATGGCGGGAATTTGCTGTGA | GCACTGCAGCAGCGGCTGATTTTTCTTCAAAT |
| *GhPP2CA17* | Gh_D05G1309 | CGCGGATCCATGGAAGAGATGTCTCCGGC | GCACTGCAGTGTTTTGCTCTTGAACTTCCTTTGAGCT |
| *GhPP2CA18* | Gh_D05G3907 | CCGGAATTCATGGCGGAGATCTGTTACGG | GCACTGCAGCGTGGCTCTCCTTAGATCCA |
| *GhPP2CA19* | Gh_D06G0657 | CCGGAATTCATGGAACCTCCACGGGAAGA | GCACTGCAGGTTGAAGAGAAGGGATTGGATTAGGT |
| *GhPP2CA24* | Gh_D12G2508 | CCGGAATTCATGACGGAGGTTTATAGGAGAA | GCACTGCAGACTTTTCTGTAGATCAACAACTA |
| *GhPP2CA25* | Gh_D13G0199 | CCGGAATTCATGGCTGGAACTTACTGTGGA | GCACTGCAGATTCGCCTGTTGATTTTTCTTTAA |
| *GhPYL2-2D* | Gh_D08G2587 | CCGGAATTCATGGACTCAGCGGAGCCACC | CGCGGATCCATCATGTCCATGAACTGAACCG |
| *GhPYL6-2A* | Gh_A06G1418 | CCGGAATTCATGCCTTCCTCTTTGCAGCTC | CGCGGATCCGGGAGATGATGACAATGATT |
| *GhPYL9-2A* | Gh_A11G0870 | CCGGAATTCATGAACGGTGGTGATGCTTAC | CGCGGATCCGTATCCGTTGATAGGCTCTG |
