# Supplementary figures and images for: Genome-wide characterization and expression analysis of PP2CA family members in response to ABA and osmotic stress in Gossypium
Source: PeerJ. 2019 Jun 14;7:e7105. doi: 10.7717/peerj.7105 (PMC6573834; doi:10.7717/peerj.7105)

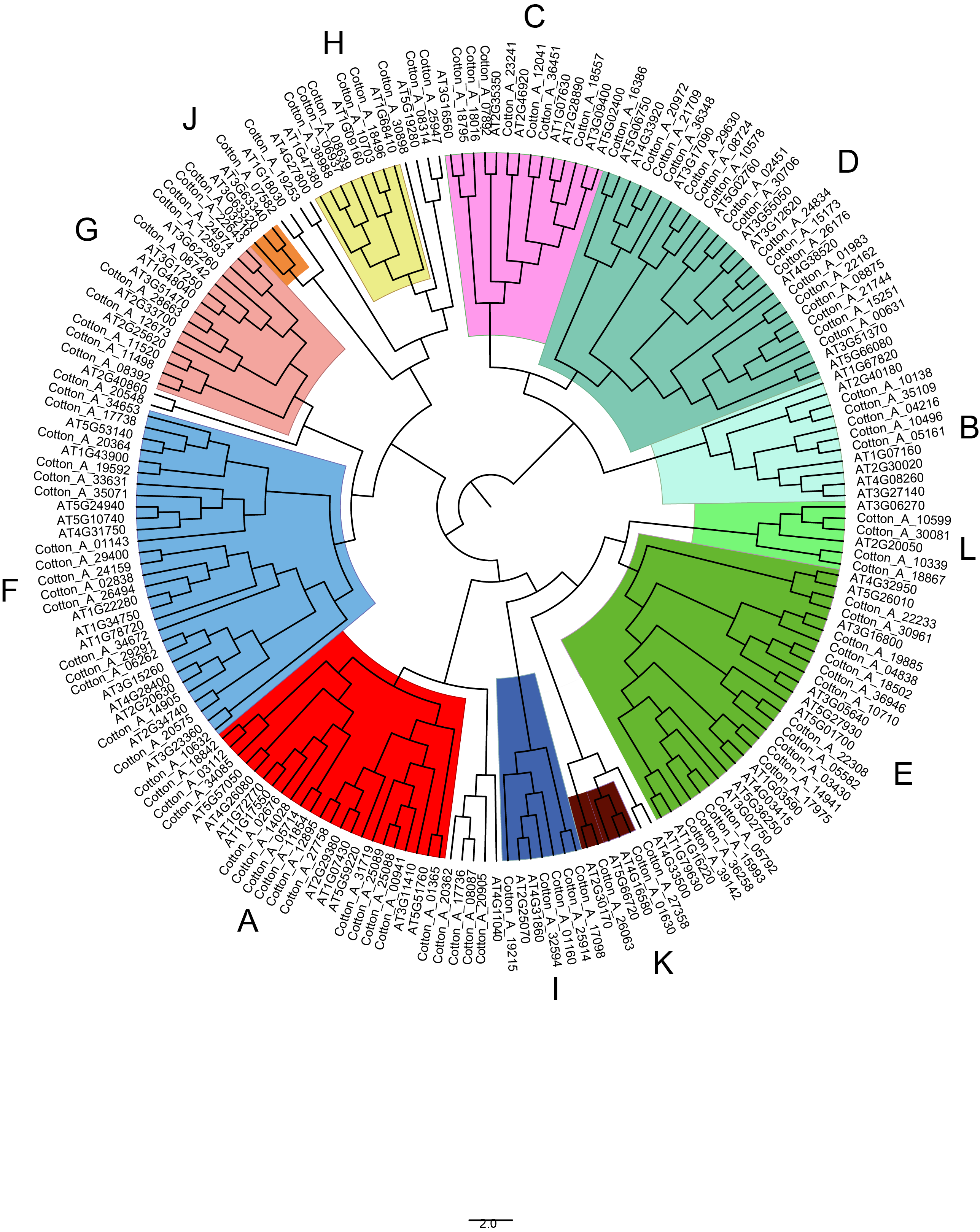

Supplement: Supplemental Information 4 — The phylogenetic tree of PP2Cs in G. arboretum and A. thaliana were generated by IQTREE server using the maximum likelihood with Dayhoff model. The PP2Cs were clustered into 12 clades (A-L) being indicated by different colors. [file peerj-07-7105-s004.png]

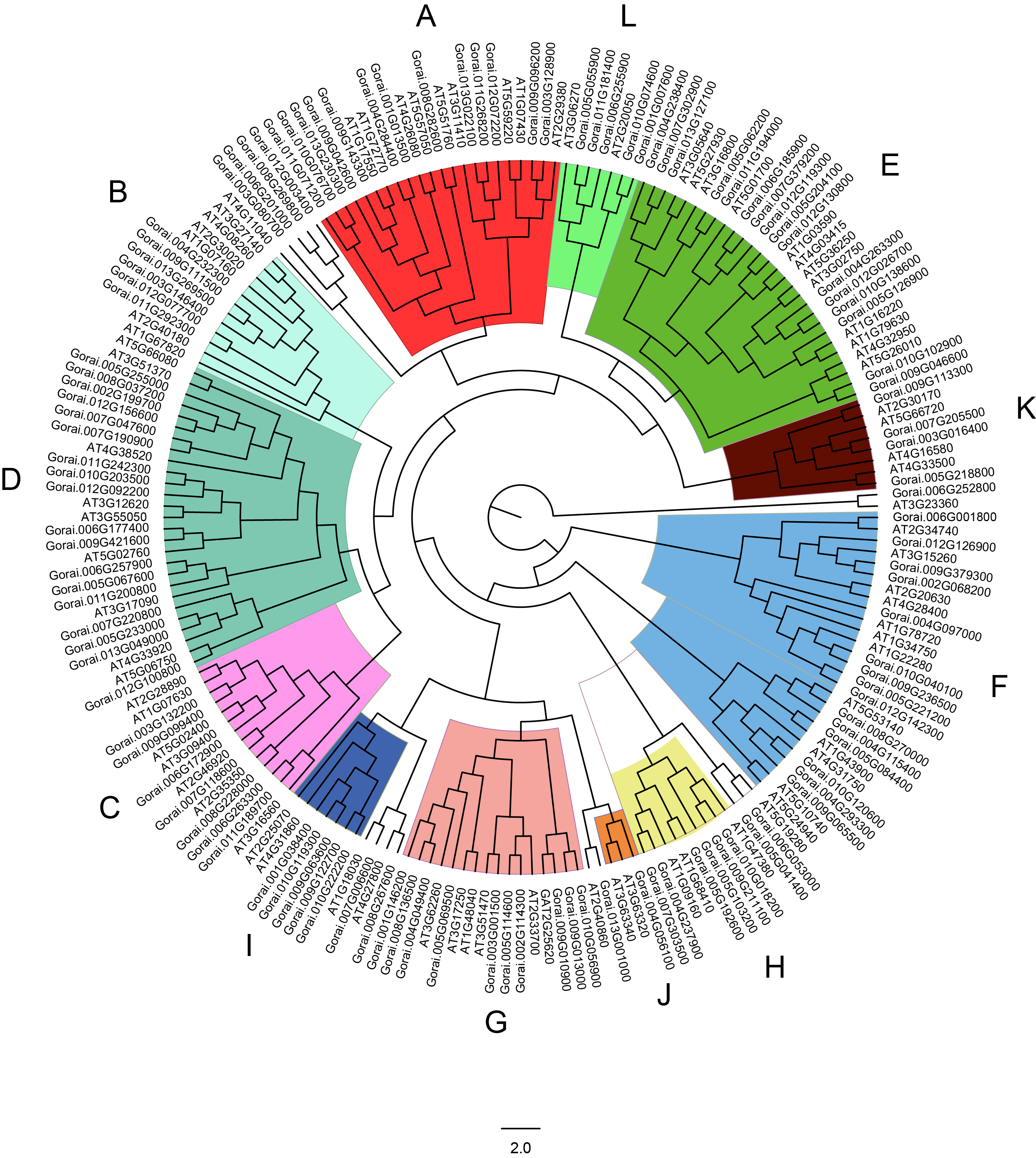

Supplement: Supplemental Information 5 — The phylogenetic tree of PP2Cs in G. raimondii and A. thaliana were generated by IQTREE server using the maximum likelihood with Dayhoff model. The PP2Cs were clustered into 12 clades (A-L) being indicated by different colors. [file peerj-07-7105-s005.png]

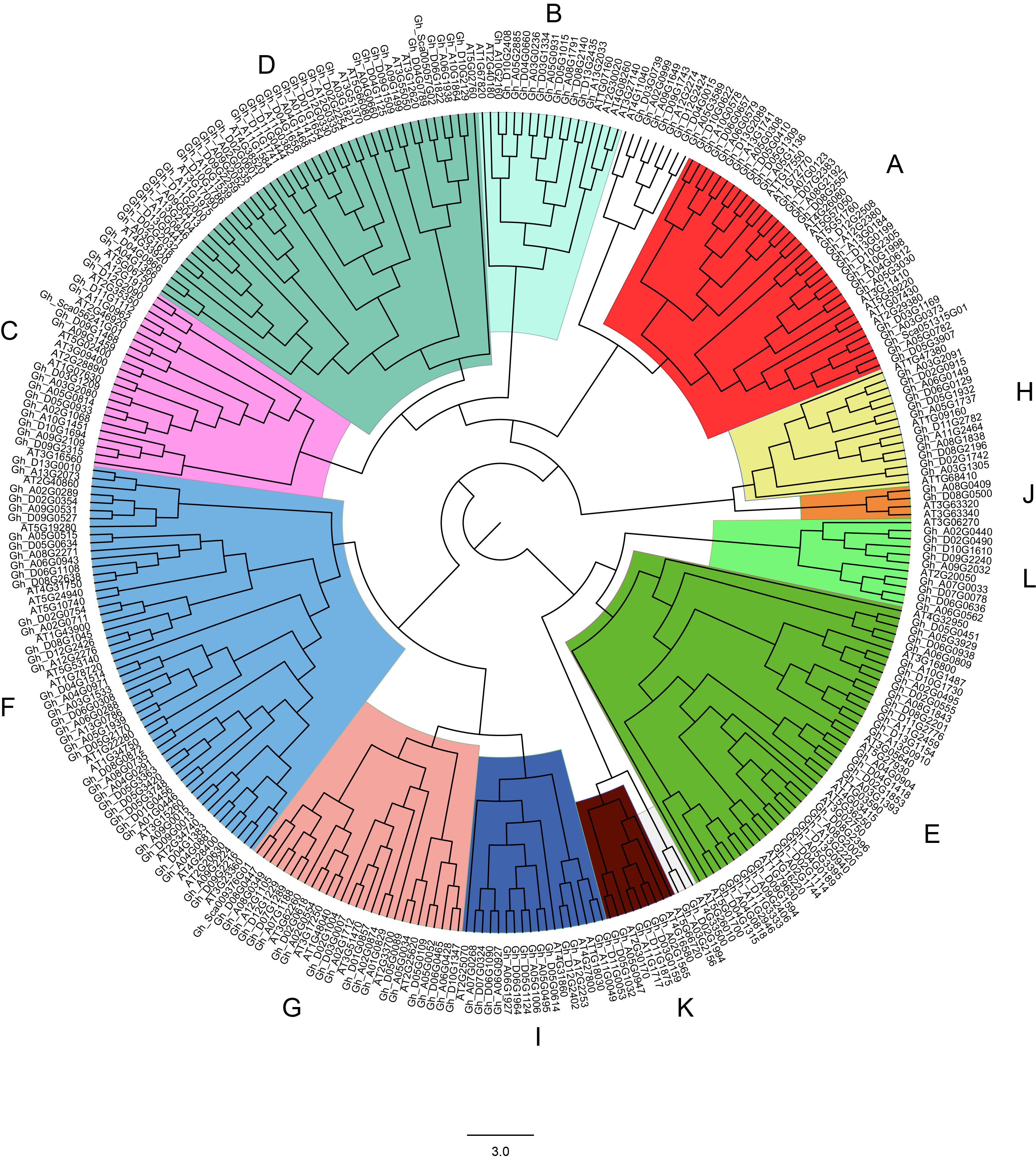

Supplement: Supplemental Information 6 — The phylogenetic tree of PP2Cs in G. hirsutum and A. thaliana were generated by IQTREE server using the maximum likelihood with VT+F+G4 model. The PP2Cs were clustered into 12 clades (A-L) being indicated by different colors. [file peerj-07-7105-s006.png]

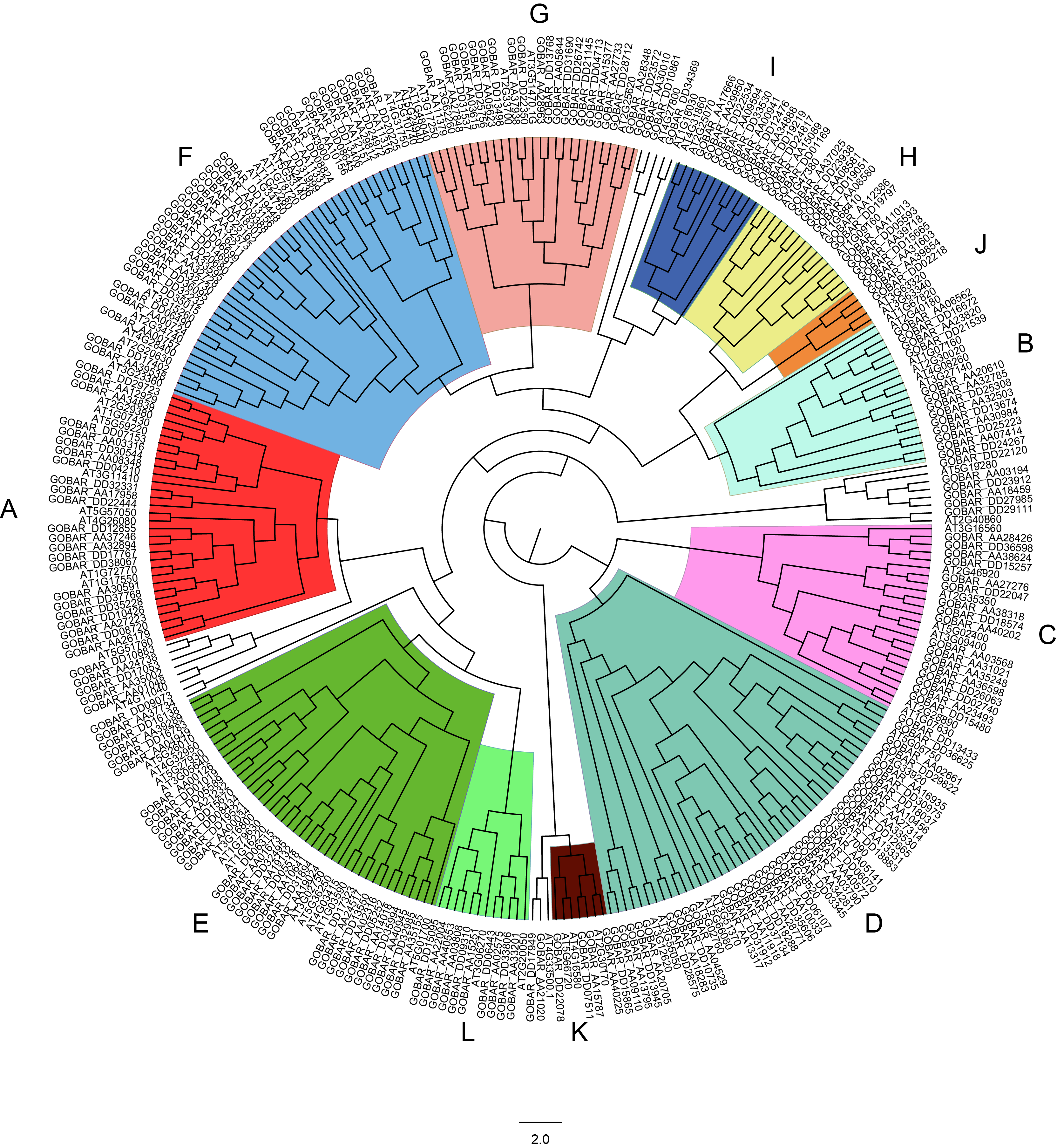

Supplement: Supplemental Information 7 — The phylogenetic tree of PP2Cs in G. barbadense and A. thaliana were generated by IQTREE server using the maximum likelihood with VT+F+G4 model. The PP2Cs were clustered into 12 clades (A-L) being indicated by different colors. [file peerj-07-7105-s007.png]
